# A Comparative Study of Physical and Chemical Processes for Removal of Biomass in Biofilters

**DOI:** 10.3390/molecules16086927

**Published:** 2011-08-15

**Authors:** Sergio Odín Flores-Valle, Omar Ríos-Bernÿ, Jorge Chanona-Pérez, Tomas Fregoso-Aguilar, José A. Morales-González, Oscar Jesús Prado-Rubianes, Rafael Herrera-Bucio, Pablo López-Albarán, Ángel Morales-González, Vicente Garibay-Febles, Enrique Godínez Domínguez, Christian Kennes, Ma. Carmen Veiga-Barbazán, Jorge Alberto Mendoza-Pérez

**Affiliations:** 1 Laboratorio de Catálisis y Materiales, ESIQIE-Instituto Politécnico Nacional, Zacatenco, 07738 Mexico, D.F., Mexico; E-Mails: sergioodin@hotmail.com (S.O.F.-V.); bernyke@gmail.com (O.R.-B.); 2 Departamento de Ingeniería Bioquímica, Escuela Nacional de Ciencias Biológicas, Instituto Politécnico Nacional, Prolongación de Carpio y Plan de Ayala s/n, Unidad Profesional Lázaro Cárdenas, Col. Casco de Santo Tomás, 11340, México, D.F., Mexico; E-Mail: jorge_chanona@hotmail.com; 3 Departamento de Fisiología, Escuela Nacional de Ciencias Biológicas, Instituto Politécnico Nacional, Av. Wilfrido Massieu s/n Unidad Profesional Adolfo López Mateos, Zacatenco, 07700, México, D.F., Mexico; E-Mail: fisiobiologo@hotmail.com; 4 Área Académica de Farmacia, Instituto de Ciencias de la Salud, Universidad Autónoma del Estado de Hidalgo Ex-Hacienda de la Concepción, Tilcuautla, 42080 Pachuca de Soto, Hgo, Mexico; E-Mail: jmorales101@yahoo.com.mx; 5 Aeris Environmental Technologies, Edifici Eureka, Campus de la Universidad Autónoma de Barcelona, 08193 Barcelona, Spain; E-Mail: o.prado@aeristec.com; 6 Instituto de Investigaciones Quimicobiológicas, Universidad Michoacana de San Nicolás de Hidalgo, Edif. B-1, Ciudad Universitaria, Francisco J. Mujica s/n, 58066 Morelia, Michoacán, Mexico; E-Mail: rhbucio@umich.mx; 7 Facultad de Ingeniería en Tec. de la Madera, Universidad Michoacana de San Nicolás de Hidalgo, Edificio "D" Planta Alta Ciudad Universitaria, Francisco J. Mujica s/n, 58066, Morelia, Michoacán, Mexico; E-Mail: plopez@umich.mx; 8 Escuela Superior de Cómputo, Instituto Politécnico Nacional, Av. Juan de Dios Bátiz s/n esquina Miguel Othón de Mendizabal. Unidad Profesional Adolfo López Mateos, 07738, México, D.F., Mexico; E-Mail: anmorales@ipn.mx; 9 Laboratorio de Microscopía Electrónica de Ultra Alta Resolución (Lameuar), Instituto Mexicano del Petróleo, Eje Central Lázaro Cárdenas Norte 152 Col. San Bartolo Atepehuacan, 07730, México D.F., Mexico; E-Mail: vgaribay@imp.mx; 10 Departamento de Estudios para el Desarrollo Sustentable de Zonas Costeras, Centro Universitario de la Costa Sur, Universidad de Guadalajara, V. Gómez Farías 82, San Patricio-Melaque 48980, Jalisco, Mexico; E-Mail: egodinez@gmail.com; 11 Departamento de Química Física e Enxeñería Química I, Facultade de Ciencias, Universidade da Coruña, Campus da Zapateira s/n, 15071 A Coruña, Spain; E-Mails: ckennes@udc.es (C.K.); mveiga@udc.es (M.C.V.-B.); 12 Departamento de Ingeniería de Sistemas Ambientales, Escuela Nacional de Ciencias Biológicas, Instituto Politécnico Nacional, Av. Wilfrido Massieu s/n Unidad Profesional Adolfo López Mateos, Zacatenco, 07700, México, D.F., Mexico

**Keywords:** downward flow, biofilter, backwashing, air sparging, biofilm, clogging, batch assay, filter bed, biomass control

## Abstract

After 6 months of operation a long-term biofilter was stopped for two weeks and then it was started up again for a second experimental period of almost 1.3 years, with high toluene loads and submitted to several physical and chemical treatments in order to remove excess biomass that could affect the reactor’s performance due to clogging, whose main effect is a high pressure drop. Elimination capacity and removal efficiency were determined after each treatment. The methods applied were: filling with water and draining, backwashing, and air sparging. Different flows and temperatures (20, 30, 45 and 60 °C) were applied, either with distilled water or with different chemicals in aqueous solutions. Treatments with chemicals caused a decrease of the biofilter performance, requiring periods of 1 to 2 weeks to recover previous values. The results indicate that air sparging with pure distilled water as well as with solutions of NaOH (0.01% w/v) and NaOCl (0.01% w/v) were the treatments that removed more biomass, working either at 20, 30 or 45 °C and at relatively low flow rates (below 320 L h^−1^), but with a high biodegradation inhibition after the treatments. Dry biomass (g VS) content was determined at three different heights of the biofilter in order to carry out each experiment under the same conditions. The same amount of dry biomass when applying a treatment was established so it could be considered that the biofilm conditions were identical. Wet biomass was used as a control of the biofilter’s water content during treatments. Several batch assays were performed to support and quantify the observed inhibitory effects of the different chemicals and temperatures applied.

## 1. Introduction

Volatile Organic Compounds (VOCs) are characterized by their tendency to accumulate and persist in the environment due to their resistance toward biodegradation [1]. Their main emission sources are anthropogenic and are directly related to industrial growth. In a large number of countries the discharge of VOCs to the environment is not properly controlled, therefore more stringent regulations for the control of emissions have appeared [2]. Elimination of VOCs from a gaseous stream can be carried out by several processes such as absorption, adsorption, incineration, and biological reaction (biotransformation) [3,4]; biofilters are included in the last category.

Biofilters work by passing a humid polluted air stream through a packed bed that is used to support microorganisms that mineralize the toxic compound [5]. Among all the different strategies used for the elimination of those compounds, biodegradation processes are often preferred. Such processes are advantageous regarding both their level of effectiveness and their cost [6,7].

For a long-term continuous operation of biofilters exposed to a high load of pollutant(s), it is essential to prevent occlusions and preferential flow because of the excessive growth of the biofilm, plugging ascribed to microorganisms, and breaking up of the bed material [8,9]. In pursuing such an objective, a few processes have been tested and reported, thus allowing the development of economic and efficient strategies in order to eliminate and to prevent the accumulation of excessive biomass and clogging materials [10,11,12]. The design of treatments is focused in reaching high and constant elimination efficiency during long periods [13]. Previous works on biomass control have shown how an excess of biomass might decrease the performance of long-term gas biofilters [14] and the application of strategies in order to reduce the occlusion problem while a gas biofilter is operating [15].

In the present study, a comparison is performed between several techniques suitable for removing biomass from a biofilter operating continuously for more than one year. Determination of the most viable methods from a technical (and economic) point of view was based both on the amount of separated biomass and on the process effect on the biofilter operation. The studies are completed with a series of batch assays in order to verify the effect of chemicals and different temperatures, used in the backwashing and air sparging procedures, over the biodegradation efficiency of biofilm samples.

## 2. Results and Discussion

### 2.1. Water-Filling/Draining, Air Sparging and Backwashing Control Treatments with Distilled Water

**Figure 1:**
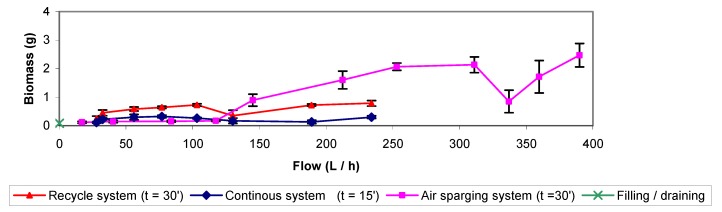
Backwashing (recycle and continous system), air sparging and filling / draining with distilled water at room temperature.

Figure 1 presents the data of air sparging and backwashing (recycle and continuous system), applying various flows either of water or air. Just one result is presented for the water filling/draining treatment since there was no applied flow. For this treatment, the amount of removed biomass was low in comparison with other methods, *i.e.* less than 0.1 g VSS with 2 L distilled water used during treatment. For the cases of backwashing and air sparging, the results indicate that the amount of biomass removed depends partially upon the applied flow either of water or air. When applying higher flows, higher amounts of biomass are removed. As can be seen, air sparging was the most effective treatment, followed by backwashing and then water-filling/draining with a nutrient solution or pure water. The order of effectiveness can be explained with basis on the magnitude of turbulence and friction forces because these are always higher for airflow than for water flow. In air sparging experiments performed at 337 and 359 L h^−1^ lower amounts of biomass were removed if compared with other results in the 300 to 400 L h^−1^ range. A possible explanation can be suggested; it was observed that before each treatment reported, the pressure drop throughout the biofilter was between 28.44 and 113.75 Pa, except for experiments at 337 and 359 L h^−1^, where the pressure drops before treatment reached values of 381.08 and 227.51 Pa, respectively. This observation suggests that the efficiency of biomass removal decreases at high pressure drops in the biofilter.

**Figure 2:**
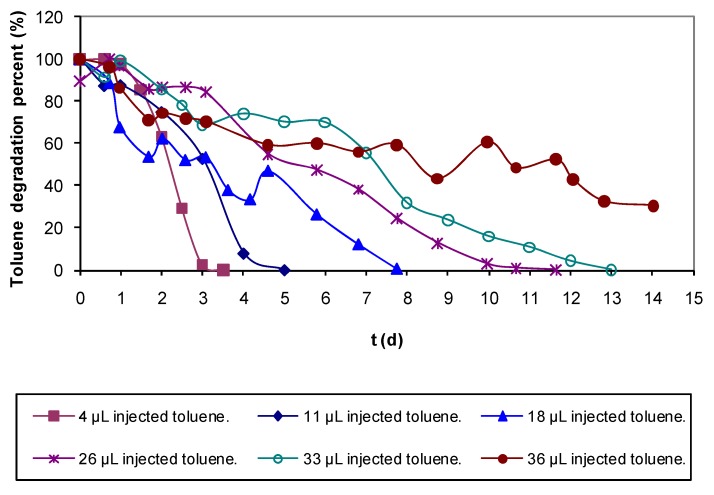
Batch assays performed to study biodegradation rates for different toluene total injected volumes at 20 °C and with 0.3 g VSS L^−1^ giving an optical density of 0.70 *A* (all the assays by duplicate).

Batch assays were performed with varied toluene concentrations. Results in Figure 2 show that biodegradation rate is inversely proportional to toluene concentration, and the microbial culture was able to completely eliminate up to 33 μL of injected toluene (90 mg L^−1^ related to the total vial volume). Furthermore, these assays were used as control for the experiments performed either at high temperatures or in presence of chemicals.

Results discussed in the following paragraphs demonstrate that higher biomass removals are attained when adding different chemicals to the treatments described in the previous section. However, should be noted that the use of chemicals increases the treatment costs.

### 2.2. NaOH Backwashing and Batch Assays

**Figure 3:**
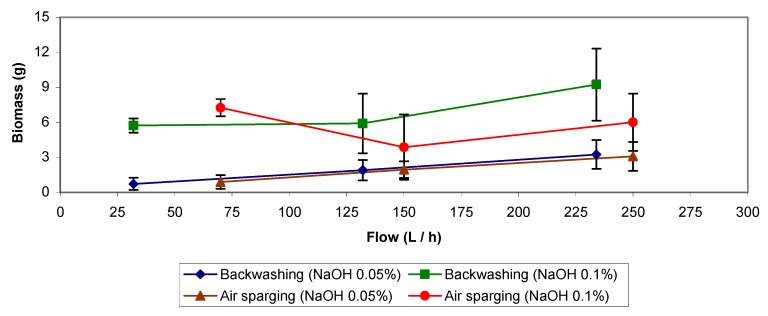
Backwashing and air sparging applying NaOH solutions.

**Figure 4:**
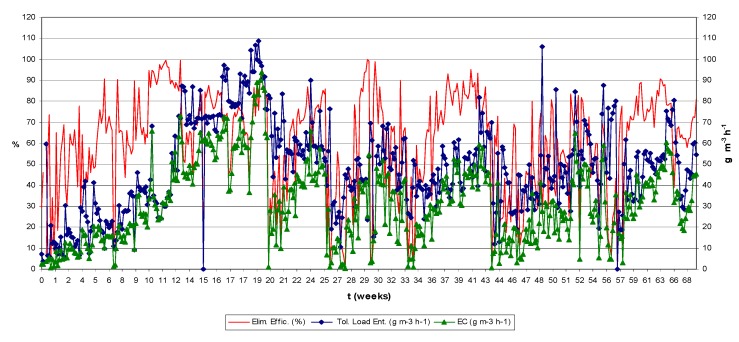
Biofilter elimination capacity and removal efficiency. I ) Filling/draining with water or nutrient sol. II-a) Water-backwashing (20 °C). II-b) Water-air sparging (20 °C). III-a) Air sparging with NaOH sol (fungus growth). III-b) Backwashing with NaOH sol (fungus growth). IV-a) Backwashing with NaOCl sol. IV-b) Air sparging with NaOCl sol. V-a) Water-backwashing at high temperatures and first manual stirring (time signal with arrow). V-b) Water-air sparging at high temperatures (fungus growth). VI) Different treatments applied. VII) Different treatments applied and second manual stirring (time signal with arrow). VIII) Backwashing with cationic detergent sol. (HTAB). IX) None treatment applied.

Results in Figure 3 for the treatments applying backwashing with NaOH solutions indicate that this is an effective but too aggressive method. Special care was taken in rinsing the filter bed after treatment in order to avoid high pH increases. In spite of all the precautions taken after carrying the treatment out, relatively large amounts of VSS were measured both in the residual and rinsing water in comparison with the treatment using distilled water only, and the biofilter presented a low biological activity compared to values reached before the treatment (See Figure 4, from week 12 to week 20). The elimination capacity dropped below 40 g m^−3^ h^−1^ at a load of 65 g m^−3^ h^−1^, giving elimination efficiency below 62% and needing a period of one week to recover. Also, during this event a fungal culture appeared (end of period IIIb), whose effect added to the one of the NaOH, apparently increased the inhibition of the biodegradation activity in the biofilter.

After treatment, the biofilter was rinsed until a neutral pH was reached, the pH after the 0.05% NaOH treatment left the bioreactor working at pH 7.5–7.8 when the usual operative pH of the system is 3.5–4. When working with higher flows, higher amounts of biomass could be removed. Larger amounts of biomass were also eliminated at the highest NaOH concentration (0.1%) but foaming problems appeared. Cox and Deshusses [11] as well as Weber and Hartmans [16] also used caustic for biomass control and already warned about foam formation when working with NaOH concentrations above 0.1%. The biofilter was submitted to a high basicity shock (pH increased to 9–9.5) and it was necessary to rinse abundantly in order to reach neutrality, unless the rinsing water was previously acidified. At the end of this treatment another fungus may be similar (but not identified) to the one that appeared in the experiment with low NaOH concentration, grew up in the biofilter bed and the elimination capacity did not exceed 10 g m^−3^ h^−1^ with a toluene load around 30 g m^−3^ h^−1^, giving an elimination efficiency of 30% (Figure 4). Again, the effect of using increasing flows resulted in a slight increment in the amount of removed biomass.

**Figure 5:**
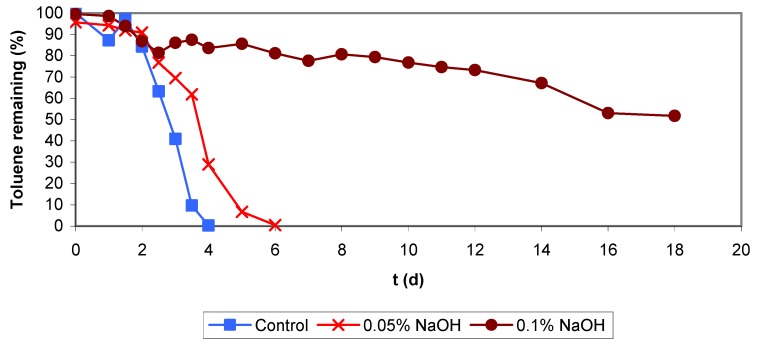
Batch assays with NaOH and toluene (11 µL volume injected).

Comparing the results from both experiments it is quite obvious that when applying higher NaOH concentrations, larger amounts of biomass could be removed, but the repercussions of this aggressive treatment on the biological activity should be considered and will be discussed. Therefore two batch assays were undertaken in presence of NaOH. In both assays the same toluene concentration had been added (toluene volume injected was ll μL and the initial pH were between 6.8–7.1 and 9.2–9.3 for 0.05% and 0.1% NaOH, respectively. Figure 5 shows data obtained from the batch assays and as it can be seen, the biodegradation activity was inhibited at the higher NaOH concentration because of the pH increase above 9.0, but also in the lower NaOH concentration the biomineralization rate was lower than in the control test without NaOH.

### 2.3. Air Sparging with NaOH Solutions

Results of this treatment also are presented in Figure 3. As in the case of backwashing, the data of air sparging experiments with 0.05% (w/v) NaOH solution indicate that when the air flow was increased, higher amounts of biomass were removed.

Data obtained for the treatment with 0.1% (w/v) NaOH solution do not follow the tendency described. After applying a flow of 70 L h^−1^ the amount of removed biomass decayed at the following flow (150 L h^−1^) and then increased at the last flow applied (250 L h^−1^). The explanation might be that when the experiment at the lowest flow (70 L h^−1^) was performed, the biofilter presented a low pressure drop of about 28.44 Pa. In the following experiments with this treatment, the pressure drop was higher (above 568.77 Pa) and the obtained data confirms our explanation, because it was observed that each time a high pressure drop was reached the treatments applied were less efficient than when the biofilter was operating at a lower pressure drop. Concluding that it is advisable not to wait until a high bed compaction is reached before applying a specific chemical or physical treatment. Also, the concentration of the NaOH solution had repercussions in the results, being more biomass eliminated with the 0.1 % (w/v) solution than with 0.05% (w/v). Same result observed than for the backwashing treatments. 

In the biofilter, foaming and a high pH were the main problems after applying this treatment. When working with the 0.1 % (w/v) NaOH solution, the biofilm reached a high final pH of 9.2–9.8 which is inhibitory, and it was necessary to rinse the filter bed with distilled water until slightly acidic conditions were again reached, as before the treatment. This was not needed for the experiments with the 0.05% (w/v) NaOH solution, because after the treatment, the final pH was about 7.4–7.8 which was acceptable for a satisfactory reactor performance. Nevertheless, the biofilter also presented inhibition of its elimination capacity and efficiency. As with NaOH backwashing using both concentrations (0.05 and 0.1%), the biodegradation activity was inhibited and the elimination capacity fell down below 30 g m^−3^h^−1^ with a toluene load of 65 g m^−3^h^−1^ and an elimination efficiency of 40% (Figure 4).

Furthermore Figure 3 compares both treatments with NaOH solutions, air sparging and backwashing, showing that the most effective for removal of biomass is the first one. This was concluded because the amount of biomass removed with air sparging treatment despite the less volume of NaOH solution and rinsing water used (5 L), can be compared to the amount removed by backwashing treatments using in total 9 L NaOH solution and rinsing water. Also it can be seen in Figure 4 that air sparging experiments performed between week 20 to week 22 present more inhibition of the biodegradation activity (low efficiency and elimination capacity) than backwashing (from week 22 to week 27). The falling down of the elimination capacity and the efficiency at the end of these treatments was increased probably because of fungal growth (end of period IIIa). The effect of the fungus seems to be added to the effect of the NaOH solutions. This conclusion has been supported with the results obtained in the batch assays performed in presence of the fungus/biomass inoculum and theoretically considering that after rinsing the biofilter with distilled water there is no excessive NaOH remaining, this comment is based on the biofilter final pH (7.4–7.8). Also, as the days go by, remaining NaOH could be eliminated when draining the excess of water accumulated in the biofilter from the wet biomass and the condensed air humidity.

### 2.4. Backwashings and Batch Assays with NaOCl

**Figure 6:**
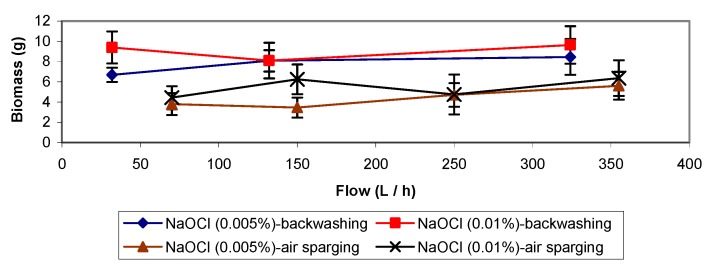
Backwashing and air sparging treatments with NaOCl solutions.

Good results could be expected with NaOCl taking into account its strong germicidal effects, and considering the data from Cox and Deshusses [11] suggesting the high efficiency of that chemical for removing biomass. Comparison of Figure 3 and Figure 6 shows that sodium hypochlorite, at very low concentrations (0.005 and 0.01%), could eliminate larger amounts of biomass than backwashing treatments adding 0.05% NaOH but gave similar results as in backwashing with 0.1% NaOH. After the treatment with 0.005% NaOCl, a drop in the elimination capacity was observed (below 40 g m^−3^ h^−1^ with a toluene load of 60 g m^−3^ h^−1^ and an elimination efficiency of 45%), needing a period of almost one week to recover an acceptable and constant biodegradation activity (elimination capacity above 40 g m^−3^ h^−1^ with a toluene load of about 50 g m^−3^ h^−1^ and an elimination efficiency of 80%, Figure 4). The elimination capacity drop and the recovery period were very similar to the reported for backwashing with NaOH solutions.

Furthermore, it should also be mentioned that the negative effects on biofilter performance were less severe. The amount of biomass removed was quite similar at the three different flow rates for a given concentration of NaOCl over the range of flow rates tested. When the concentration of hypochlorite was increased from 0.005% to 0.01%, slightly higher amounts of biomass could be eliminated. The pressure drop in the biofilter was always below 113.75 Pa when applying the treatment, except for the experiment with 0.01% NaOCl and a flow rate of 132 L h^−1^ (pressure drop above 170.63 Pa) which does explain the slightly worse result obtained in that case.

The germicidal effect of 0.01% NaOCl concentration was still important, carrying as consequence almost the total inhibition of the biodegradation capacity of the biofilter and a very lingering period time (longer than for the case of the NaOH) to recover the previous elimination capacity of the bioreactor.

**Figure 7:**
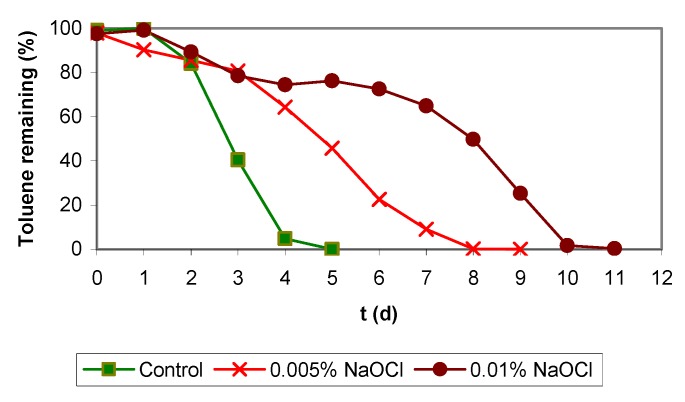
Batch assays with NaOCl and toluene (11 µL volume injected).

Batch assays support the above comments on the strong germicidal effect of NaOCl solutions and the negative effect on the elimination rate of toluene. The difference between the control experiment and the assay with the lowest NaOCl concentration (0.005%, w/v) was of about 3–4 days. Being more notorious than the difference between both assays with NaOCl (0.005% and 0.01%, w/v) which was about 2 days (Figure 7). In batch vials with higher concentrations than 0.01% (w/v), the alkylbenzene simply could not be eliminated. Other concentrations tested were 0.025, 0.05 and 0.1% (w/v) but all the batch assays presented total inhibition of the biodegradation activity (data not shown).

### 2.5. Air Sparging with NaOCl Solutions

NaOCl solutions of 0.005 and 0.01% (w/v) were used with four different flows, 70, 150, 250 and 355 L h^−1^, at 20 °C (Figure 6). Higher amounts of biomass were removed compared to the results of the treatments with 0.05% NaOH solutions. On the contrary the results were relatively similar or even slightly worse than in the assays with 0.1% NaOH. In this experiment, the amounts of biomass removed were a little higher when increasing the flow or the NaOCl concentration (Figure 6). Results that do not fit with the mentioned statement can be explained as an effect of high pressure drop in the biofilter (above 227.51 Pa, as average value), resulting in a high compaction of the biofilm and channelling. The best results were obtained with 0.01% NaOCl (w/v), removing 6.35 g VSS at a flow of 355 L h^−1^. When working with a 0.005% NaOCl solution and a flow of 355 L h^−1^, 5.6 g VSS was the largest amount of biomass eliminated. At the end of the experiments, biofilter pH was between 4.6 and 5.4. Its elimination capacity was below 20 g m^−3^h^−1^ with a toluene load of 50 g m^−3^h^−1^ and an elimination efficiency of 40% (Figure 4). The biofilter required almost two weeks to again reach its previous performance. Such q long period for re-establishment, compared to the NaOH treatments, can be explained because of the high germicidal effect of NaOCl added to the disruptive effects over the biofilm, when the filter bed was broken up by shaken and stirring caused by the air sparging process.

### 2.6. Backwashing Strategy Applying a Cationic Detergent: Hexadecyltrimethylammonium Bromide (HTAB)

**Figure 8:**
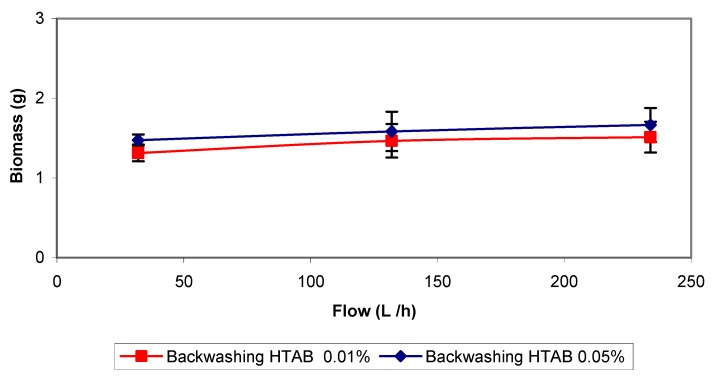
Backwashing treatment with cationic detergent (HTAB).

Summarized in the literature [16,17,18,19], it is known that most microorganisms present negative charges in their cell wall, integrating electrostatic forces that help them attach to the biofilm structure, jointly with polyanionic matrix polymers (polysaccharides) and metal bridge cations. In order to eliminate the charge interactions the next strategy consisted in using a cationic detergent, an ammonium salt. The experimental results are shown in Figure 8. Two concentrations (0.01 and 0.05%) and three different flows were tested. As observed in the curves, very similar and relatively low amounts of biomass were removed with both concentrations. The flow rate appeared not to affect the results. Foaming was the major problem and it was necessary to rinse several times the filter bed until the recycle water stopped forming foam. The pH was not significantly altered after rinsing the biofilter, and remained around 4.5–5.5. The bioreactor presented inhibition after applying this treatment but it was not as strong as in the treatments with NaOH or NaOCl.

**Figure 9:**
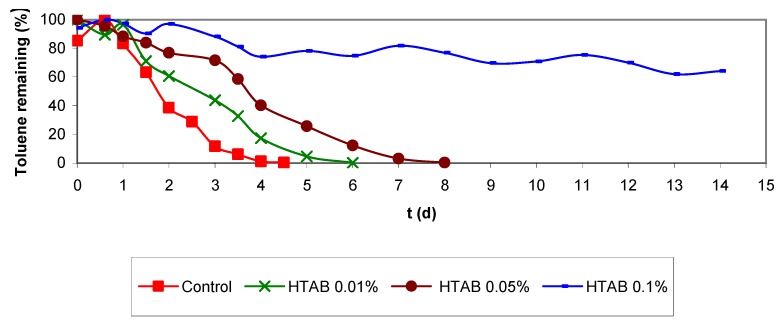
Batch assays with cationic detergent (HTAB) solutions and 11 µL toluene volume injected.

Batch assays with HTAB were performed using three surfactant concentrations and 11 μL of injected toluene. In the experiments with cationic detergent concentrations of 0.01 and 0.05% as used in the biofilter, the biodegradation rate was slightly lower than in the control assays (Figure 9), indicating an inhibition of the biodegradation activity. In the assays performed with the highest HTAB concentration (0.1%), the biodegradation activity was totally inhibited. These data show that the inhibitory effect is directly related to the detergent concentration. In Figure 9, it can also be observed that the biodegradation rate was higher for batch assays with 0.01% detergent than for experiments with 0.05% HTAB concentration. However, control assays performed with 11 μL toluene but without cationic detergent, presented the highest biodegradation rate. The results indicate that the concentrations used in biofiltration experiments did not cause total inhibition of the microbial culture, but were high enough to inhibit partly biodegradation.

### 2.7. Backwashing Strategy and Batch Assays at High Temperatures

**Figure 10:**
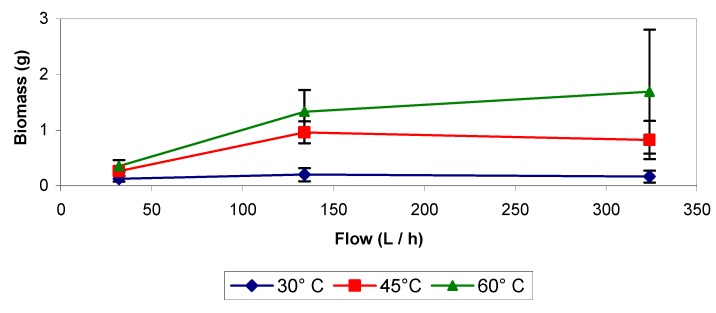
Backwashing applied at different temperatures and flow rates.

When carrying out backwashing with distilled water and different combinations of temperatures and flows, little biomass was removed at 30 °C (Figure 10) and the results were similar to the data obtained at 20 °C (Figure 1). At 45 and 60 °C, the eliminated biomass was larger than at 30 °C, but among both temperatures a very notorious difference was not observed, although the amount of biomass eliminated was higher at the highest temperature (Figure 10), even more the variation in the amount of removed biomass was very large. In the experiment at 60 °C with a flow of about 324 L h^−1^ (Figure 10), less biomass was removed at the highest pressure drop, about 455.02 Pa, of the biofilter due to the presence of more compacted biomass and channeling. It was also observed that treatments performed with pure distilled water at room temperature and high temperatures presented less inhibition than the treatments applying chemicals, but the amounts of removed biomass were lower in the first case. This can be related to the effects that increase the pressure drop in the bioreactor (biosupport compaction and channeling). Results indicated that the higher the pressure drop was, the lower the amount of biomass removed was when the first treatments were applied, but data obtained with the treatments applying chemicals showed that it was possible to remove high amounts of biomass even when the biofilter presented pressure drops lower than 284.39 Pa, but not above. When the pressure drop reached values higher than 284.39 Pa (usually between 341 and 568 Pa) it was necessary to apply the treatment several times until a low pressure drop was reached. This could be the reason why data obtained in some of these experiments presented so much variation and as low pressure drops were reached then larger amounts of biomass were removed. The results of the treatments with pure distilled water at room temperature and high temperatures showed that these were not so effective when the pressure drop was between 113.75 and 170.63 Pa, so the best results could be obtained working with these treatments if the pressure drop presents a low value but if that is not the situation it is better to apply a treatment that uses a chemical solution.

**Figure 11:**
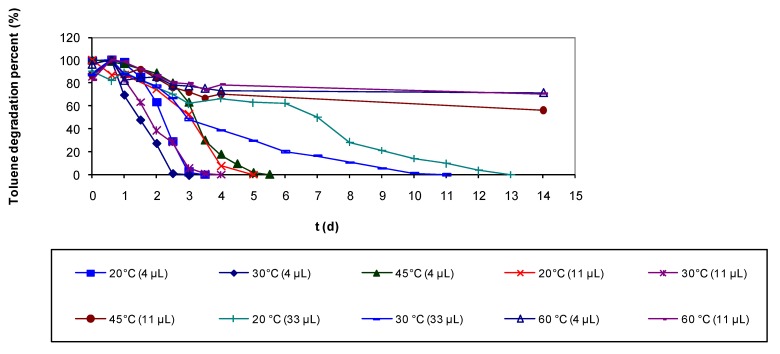
Batch assays at different temperatures and volumes of toluene.

When analyzing the results of the experiments, it was observed that the effect on biomass removed of increasing the flow at low flow rates (below 134 L h^−1^) is more significant than at higher flow rates. This can be due cause a low flow cannot remove biomass that is too soggy. Results of batch assays at high temperature are presented in Figure 11. At 30 °C it was observed that the biofilm is able to eliminate up to 36 μL of toluene, when that same concentration could not be eliminated in the control experiments at 20 °C. Also at 30 °C, the biodegradation rate for all the tested concentrations was much higher than for the controls at 20 °C (Figure 11).

At 45 °C, toluene biodegradation was only possible in the experiments with 4 μL of toluene and the degradation rate was lower compared to the control at room temperature (Figure 11). In the assays at 60 °C the microbial culture was completely inhibited and there was no biodegradation, but after two weeks experiment, the vials were treated at 20 °C and total elimination of the recalcitrant compound was achieved (Figure 11).

### 2.8. Air Sparging at High Temperatures

**Figure 12:**
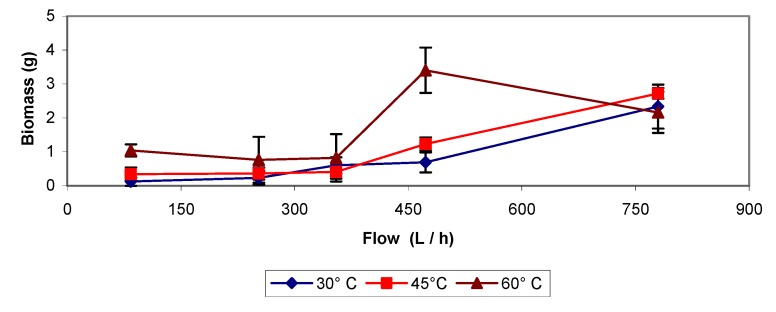
Air sparging applied at different temperatures and flow rates.

Data shown in Figure 12 indicate that as the air flow is increased, larger amounts of biomass are removed. This effect is more notorious at higher flows (above 340 L h^−1^).

By comparing data at 30 and 45 °C it seems that a temperature increase does not improve the process efficiency. Contrary to results at 60 °C where the amount of biomass removed was significantly higher than at the other two temperatures, having a high increase while working with a flow around 473 L h^−1^ and then observing a decrease at the higher flow of 783 L h^−1^ that can be explained by a high pressure drop effect mentioned above.

After the experiments applying air sparging at high temperature (end period Vb) the growth of a fungus was noticed. Its repercussion in the bioreactor operation was negative, because elimination capacity and elimination efficiency decayed (below 15 g m^−3^h^−1^ and giving a 30%) and until the fungus was not eliminated both parameters could not be re-established up to the previous values (Figure 4).

**Figure 13:**
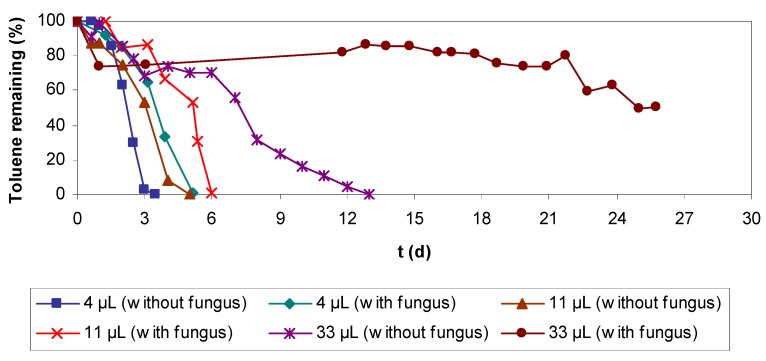
Batch assays with and without fungus (0.3 g VSS L; OD about 0.65–0.70 *A*) at 20 °C and different volumes of injected toluene.

Contrary to other reports in the literature [16] where fungus present an elevated capacity to eliminate recalcitrant compound(s), it was determined this occupation was a negative one, because the biodegradation data obtained for the biofilter during the period of permanence of the fungus until its eradication by using a fungicide, were quite irregular; the removal efficiency decayed (Figure 4) and also a high pressure drop was achieved (higher value was 2,024.80 Pa). This was also confirmed with results from a series of batch assays that were carried out with samples of fungus contaminated biolayer, having on purpose to analyse the effect of the invader organism over the biofilm biodegradation activity (Figure 13).

These batch assays were performed as described in materials and methods section but with the difference that the vials were inoculated with biofilter biolayer contaminated with fungi, previously an inoculum used to seed the vials was prepared following the procedure described also in materials and methods. Vials were inoculated to contain 0.3 g VSS L^−1^, resulting in an optical density between 0.65–0.70 *A*. For comparison of the results, previous batch assays performed with biofilter biolayer without fungi and used to study the biodegradation rate at different toluene concentration (Figure 2) were used as control experiments.

### 2.9. Manual Stirring for Removal of Biomass

Two experiments with manual stirring were performed at different times during biofilter operation (Figure 4). After applying manual stirring to the biocatalyst, it was rinsed with 3 L distilled water or with nutrient solution. Wastewater was collected and all parameters previously mentioned in the material and methods section were analyzed. Large amounts of biomass were removed but much bed material was disrupted, and it was necessary to add some to replace it with new, clean, non-inoculated material. The removed biomass was 12.3 and 10.2 g VSS for each experiment, which are much higher values than in all other cases, but as Laurenzis *et al.* [20] indicated, this is an expensive method. On the contrary, almost no depletion of the biodegradation activity was observed and the biofilter delayed no more than three days to reach previous operating values (before treatment), reaching an elimination capacity higher than 20 g m^−3^h^−1^ with a toluene load of 35 g m^−3^h^−1^ and an elimination efficiency of 70%, after the first experiment and in the second experiment the higher value reached after two days of its application was 50 g m^−3^h^−1^ of elimination capacity with a toluene load entrance of 65 g m^−3^h^−1^ and an elimination efficiency of 70%.

### 2.10. Biofilter Water Content, Total and Dry Weight

Sudden important decreases in removal efficiency and elimination capacity are due to the effect of feeding, backwashing or air sparging. The wide variation in time response of the biofilter to reach its highest removal efficiency after being submitted to the different biomass elimination processes is directly related to the type of treatment and its effects on the biolayer. Some of these effects include microbial activity inhibition, biolayer disruption, changes in the composition of the microbial community and appearance of new organisms (fungi).

**Figure 14:**
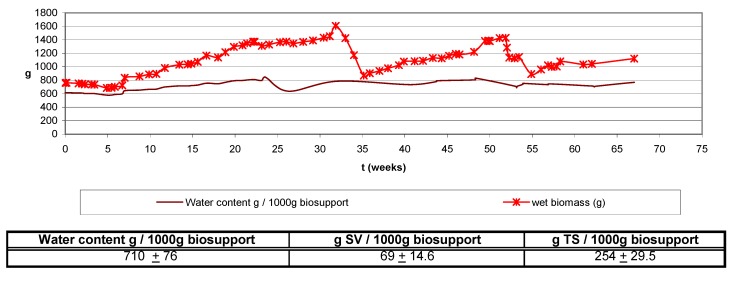
Biofilter water content and wet biomass.

**Figure 15:**
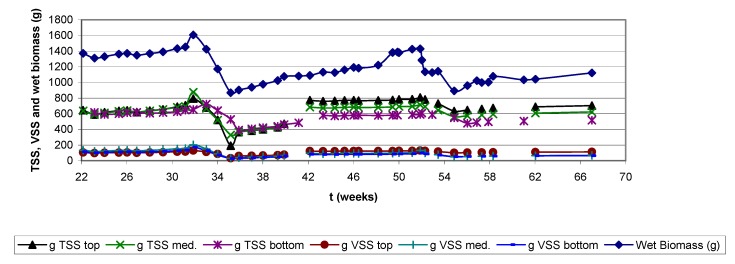
Wet biomass, VSS and TSS (both dry) at upper, medium and bottom biofilter heights.

Other authors as Cox and Deshusses [11]; Wubker *et al.* [21]; Pedersen *et al.* [22]; Weber and Hartmans [16] have reported similar effects as observed in the present research. Furthermore, in this work the time response for again reaching pre-treatment removal efficiencies also seems to be related to the loading, contrary to data reported previously [13,23], where high removal efficiencies were achieved, working with high loads, in a brief time period (hours) after being applied the treatment to remove biomass. Data obtained show that the water content of the biofilter remained between 710 ± 76 g H_2_O/1,000 g biosupport on average, during the almost 16 month experiment (Figure 14). This value was calculated as the difference between the weight of a biosupport sample, before and after drying at 105 °C. A relative constant biomass amount was observed from the results of dry weighing analysis despite the periodic increase of the alkylbenzene load, even though it is observed that biomass growth at the entrance zone was higher than at the medium and bottom zone, being the last one the least colonized (Figure 15). The reason of the uncontrolled increasing and decreasing of biomass during the almost twelve experimental months, contrary to the continuous growth data reported at the initial time of the study (t = 0; operating first months), is explained as the result of the backwashing and air sparging fluidizations applied to the bioreactor in order to limit the bacterial growth.

Furthermore, the reason why large amounts of biomass are observed at the initial time (t = 0) in Figure 15 is because the biofilter had already been operating for several months (at least 6) in a first experiment prior to this second long-term experiment. After two weeks stoppage, the biomass was not removed and the biofilter was started up with the same microbial growth, without any addition of new microbial culture. The total weight of the biofilter gives jointly information about the biomass growth and changes in the water content (wet biomass). Wet biomass data from Figure 15 is obtained by substracting from the total biofilter weight, the weight of the glass column and the clean, non inoculated inert support dry weight. It is important to mention that as the biofilter wet biomass increases constantly despite the continuous removal of biomass caused by the treatments, the dry weight results show no important increments; this can be explained as a consequence of the non- controlled amount of water that could be retained inside the biofilter.

### 2.11. Effect of High-Pressure Drop when Applying a Treatment for Biomass Removal

**Figure 16:**
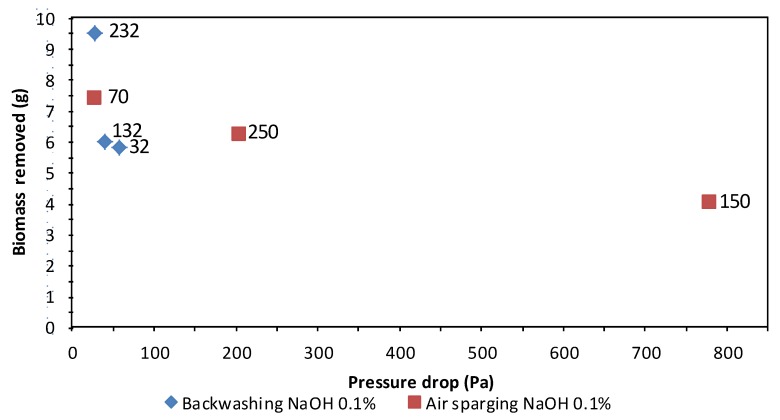
Effect of high pressure drop over the amount of removed biomass when treatments performed were applied at different flow rates. In the graphic, right hand data indicate the water or air flow rate value (L h^−1^).

Several times in this communication, the pressure drop has been mentioned when trying to give an explanation as to why the applied treatments have not removed the expected amount of biomass in accordance with the water or air backflow value and the chemical compound used to performance the treatment. It is known that pressure drop is in some way related to the compaction grade of the biopacked bed (product of water content, air flow and biomass weight) and also with channeling and partial zone clogging. Figure 16 shows a relation between the amount of removed biomass and the increment of pressure drop in the results of two treatments, indicating that it can be considered that when the pressure drop was higher, less biomass were removed independently of the treatment and the water or air backflow applied. It was observed that after applying the treatment several times (instead of increasing the water or air backflow applied) while the pressure drop was decreasing (meaning that the biopacked bed compaction and/or channeling and/or clogging were diminishing), the amount of biomass removed was higher. However, sometimes the results could never reach the expected values but it were something more reasonable. We tried to establish an interval to reach the higher efficiency of every treatment applied to remove biomass, but the interval of observed values is quite large, so average values were calculated for each treatment and those are the values mentioned above for each case. Obviously the pressure drop is related to the presence of channeling zones or biofilter partial zone clogging (not only a uniform compaction), affecting in more or less acute ways the treatments performance, so this could be the explanation to the high variability of the amounts of removed biomass and its apparently not uniform relation with the pressure drop.

## 3. Experimental

### 3.1. Biofilter Apparatus

**Figure 17:**
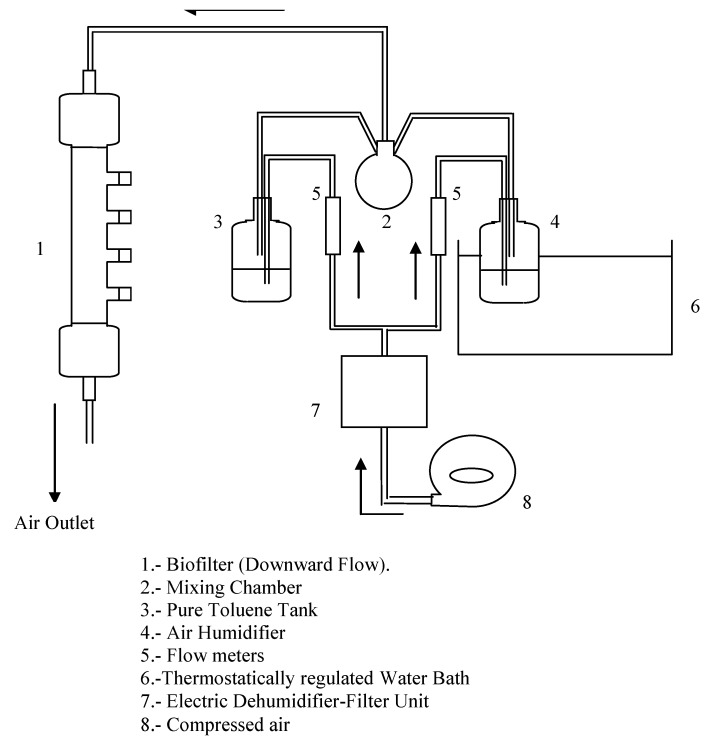
Biofilter design.

The reactor is made of a glass column with an inner diameter of 7.3 cm and an overall height of 94 cm (Figure 17). It is filled with 2.1 dm^3^ perlite grains [24]. The free sections at the column top and bottom are 22 cm and 14 cm in length, respectively. Four ports for gas sampling are located at 14.5 cm intervals. All the surfaces in contact with the contaminated air are made of glass, Teflon^TM^ or Viton^TM^. Aluminium foil was wrapped around the bioreactor in order to avoid growth of algae. Toluene was the alkylbenzene selected as the model VOC.

A flow of compressed air passes through an electronic dehumidification filter, the resulting dry air flow is divided in two, one fraction is connected to a termostated humidification tank and the other one is saturated with toluene in a stripping bottle. The flow rates of both humid and saturated streams are measured and regulated at the outlet of the humidification tank and the stripping bottle, respectively. The streams enter a mixing chamber before flowing through the biofilter in downward direction. The empty bed gas residence time (EBRT) was initially established at 56. Toluene loading (TL, g m^−3^ h^−1^), elimination capacity (EC, g m^−3^ h^−1^) and elimination efficiency (EE, %) are determined as described by Fortin and Deshusses [25].

### 3.2. Organisms and Culture Media

A defined bacterial-fungal consortium was originally used to inoculate the biofilter, obviously the microbial culture present in the reactor 1.9 years after its start-up, was more complex when compared to the original one. A nutrient solution was fed to the reactor every eight days, as described elsewhere [26]. pH was not regulated in order to gain reproducibility, acidification of the filter bed was observed. A buffer phosphate solution was added to the nutrient medium for establishing pH at 5.9 ± 0.1.

### 3.3. Experimental Set-Up

Three parameters were periodically determined in the biofilter: the wet biomass (weighting the biofilter and subtracting the weights of both the body of the reactor and the inert support), the biomass dry weight at three different reactor zones (top, medium and bottom), and the water content, which is calculated from dry weight samples and the filter bed volume.

The toluene load was gradually increased every time the removal efficiency was above 80%. The experimental methods used to eliminate excess biomass and remove clogging material were tested after operation during two years. One experiment at a time was performed every 10 days. The biofilter reached the same total weight in each occasion, excepting two experiments where mechanical removal was used.

**Figure 18:**
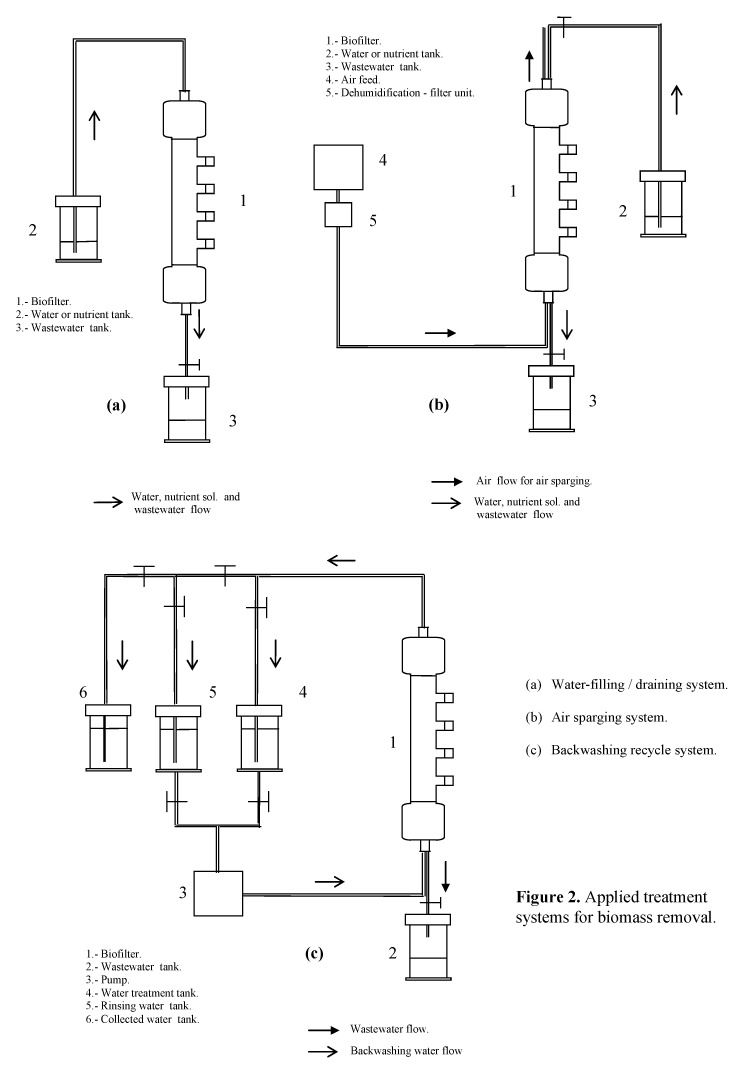
Applied treatment systems for biomass removal.

A scheme for the three main methods used for elimination of biomass is presented in Figure 18. For the water-filling/draining experiment (without any flow), the biofilter was filled with 2 L of either distilled water or nutrient solution. The effect of temperature on the elimination method was evaluated for distilled water at 30, 45 and 60 °C. Experiments performed at room temperature were used as control. Backwashing was applied as described by Rihn *et al.* [27] with the difference that 5 L water were used at room temperature (as control) and at 30, 45 and 60 °C. Several water flows were tested in an effort to verify if higher flows facilitate the scouring process of the biofilter. The procedure lasted no more than 30 minutes and at its end the biofilter was rinsed with 2 L distilled water. During the first experiments water was not circulated; although circulation was studied later in order to estimate its effect on the amount of biomass removed.

Air sparging was similar to the filling/draining experiment but an upward airflow was sparged through the filter bed. The reactor was filled with 2 L distilled water prior to air sparging; at the end of the experimentation packing was rinsed with 1 L distilled water. The effect of several flows and temperatures (30, 45, 60 °C) on the performance of this method was studied.

Chemical treatments were carried out with solutions of NaOH, NaOCl or hexadecyltrimethyl-ammonium bromide (HTAB). The solutions were used in backwashing and air sparging experiments, however the cationic detergent solution (HTAB) was omitted for air sparging because of high foaming problems observed during the backwashing experiment. All experiments were duplicated at the least. Total weight of the biofilter was measured for controlling the biomass growth in an effort to apply the treatments at similar initial microbial mass.

For NaOH, two different concentrations were tested: 0.05 and 0.1% (w/v). For treatments with NaOCl, 0.005 and 0.01% (w/v) solutions were prepared. HTAB concentrations of 0.01 and 0.05% (w/v) were used in experiments with cationic detergent solutions. Backwashing with upward flows were applied with a peristaltic pump at three different rates: 32, 132 and 234 L h^−1^. 5 L of solutions were prepared for circulation and 2 to 4 L distilled water were used to rinse the biofilter until reaching a neutral pH, allowing a fast recovery of the biofilm activity. Air sparging experiments were performed with 2 L of each solution by applying upward flows ranging from 84 to 780 L h^−1^. As in the case of backwashing, after each assay, the filter bed was rinsed with 2 or 3 L distilled water until neutral pH was reached. All the treatments were performed at room temperature and their application time never lasted more than 30 minutes.

The time the reactor delayed to reach an elimination capacity close to the value before treatment application was monitored. Periodical feeding of the bioreactor with complete nutrient solution helped to rinse residual amounts of reagents. The feeding was performed every eight days either between each experiment or in the same day after applying the treatment.

Finally, two discontinuous manual-removal treatments were applied. When clogging in the biofilter became significant pressure drops greater than 568.8 Pa were observed, resulting in a significant decrease of the elimination capacity. In this case the biofilter was temporarily stopped and the support was unpacked and mixed manually. After re-packing, the biofilter was fed with nutrient solution allowing for removal of both detached biomass and residual inert material. The broken support material was always replaced with new one.

Total wet biomass was measured and samples from different column zones were submitted to dry weight analyses. Wet filter bed material with attached biomass was calculated subtracting the weight of glass column and the rest of components of the biofilter from its total weight. By knowing the weight of the inert support volume without biomass then the weight of the wet biolayer can be calculated and the results compared with those obtained from dry weight analyses in order to determine the total water content. For each experiment, optical density determination, pH control, VSS and TSS analysis were performed for all of the collected wastewater.

### 3.4. Batch Experiments

All the batch assays were performed as described by Veiga and Kennes [28]; 315 mL vials were filled with a calculated amount of a previously prepared microbial culture and water enough to obtain a final liquid volume of 50 mL and a specific optical density. The initial biomass amount was 0.3 g VSS L^−1^ corresponding to an optical density (at 660 nm) of about 0.7 *A.* For all the assays, initial pH ranged from 5.88 to 5.91, and initial concentration of dissolved oxygen ranged from 5.25 to 5.5 mg L^−1^. Toluene was the only carbon source added. Although removal data in batch assays are reported in percentage, the toluene concentrations in the liquid and gas phases can easily be calculated using the Henry’s law constant for toluene (0.27 at 25 °C), the amount of injected toluene, and the water / air phase volumes in the vial.

The previously prepared microbial culture used as inoculum for seeding the experimental assays was obtained with 2.5 g of colonized biofilter support, seeded it in 500-mL vials with 200 mL of sterilized mineral medium. Filter-sterilized vitamin and trace mineral solutions were also added. The sealed vial was shaken at 200 rpm and room temperature. After a short time the inert support material was broken but after leaving it to settle down it was separated either by decantation or filtration using large diameter pore size filter paper (>1.4 μm). Then, 100 mL of this filtered culture were transferred to a vial containing 100 mL of the same medium described previously. Finally, a calculated amount of toluene was added to activate the elimination capacity of the microorganisms. The vials were sealed and shaken again as mentioned above. Optical density, pH, VSS and TSS were measured in the batch assays as well as in the inoculum solution.

Biodegradation assays were performed at room temperature, 30 and 45 °C, by adding 4, 11, 18, 26, 33 or 36 μL of toluene to the vial (total volume of 315 ml). Lower concentrations with 4, 11 and 18 μL of toluene were used at 60 °C. NaOH concentrations tested for batch assays were 0.05, 0.1, 0.25, 0.5 and 1% (w/v); for NaOCl 0.005, 0.01, 0.05, 0.1, 0.25% (w/v) and for hexadecyltrimethylammonium bromide (HTAB) 0.01, 0.05 and 0.1% (w/v). In the studies with chemicals a single concentration, corresponding to 11 μL of toluene, was used. Biodegradation was followed until toluene was completely eliminated. Batch experiments were also used for studying the effect over the biolayer biodegradation capacity of an unclassified external fungus that colonized the biofilter in some specific occasions, using total toluene volumes from 4 to 36 μL, at room temperature, 30, 45 and 60 °C.

### 3.5. Analytical Methods

Gas chromatography was used for toluene analysis, as described by Veiga and Kennes [28]. Samples concentrations were calculated by comparing the GC response area to the one obtained with an external standard of known toluene concentration.

Optical density was measured at 660 nm with a UV-Vis Perkin-Elmer spectrophotometer using distilled water as blank. All VSS and TSS analysis were performed after ending of the experiments. Samples from residual, recycle and rinse water were analysed in duplicate in accordance with Methods 2540C (TSS) and 2540E (VSS) of *Standard Methods* (APHA) [29]. pH values were measured using a Crison pH-meter (model 507) connected to an Ingold electrode (model U455-S7), which was calibrated at 20 °C with buffer solutions (pH 4.0 and 7.0) supplied by the manufacturer.

Dry weight analyses were performed according to method 2540G of *Standard Methods* (APHA) [29] and results were corrected for the salts present in the medium. Total biofilter weight (wet) was measured with an electronic balance (Gram, model HGS-15K). HPLC analyses of the wastewaters obtained in the treatments were performed periodically in order to verify the presence and accumulation of biotransformation products reported previously [3]. Pressure drop was measured using a Warburg manometer expressing the obtained value (cm of H_2_O) in Pa.

## 4. Conclusions

The results of this research show that as long as a biofilter needs to be supported in order to keep it working in an efficiently operational level, the treatments applied must be as harmless as possible and economically viable too. In spite of the no net cell growth conclusions of several authors [30,31,32,33] biomass growth in long-term period bioreactors, even if it is not too high, can have repercussions on the biofilter efficiency. Much of that excessive biomass does not come from the original microbial culture but it can be part of cryptic growth [10,13,21,22,34] that could or not bring benefit to the elimination capacity of the biolayer.

As it can be observed in the data, the amount of removed biomass was larger when working with the chemical combined processes than with that ones using high or room temperature pure water. Nevertheless these procedures caused the greatest damage to the biolayer and the periods to recover an operational quasi-steady state with a high elimination capacity were longer.

High temperature pure water treatments seems to be more efficient than with room temperature pure water, but the first group implicates to work with expensive processes because its higher energy cost. Finally it is obvious that air sparging gave better results compared to the backwashing process and even more against the water-filling/draining (considering that a full bed expansion was not reached). Being the last one the less efficient but the most inexpensive and *vice versa* compared to the other two treatments, both of them requiring high cost electric energy equipment (high pressure air compressors, water recycle pumps, electrical dehumidification filters and electrical water recycle systems). Recovery periods to reach again a quasi-steady state with high elimination capacity were in all the treatments free of chemical compounds, shorter than in the last mentioned.
